# MHD dissipative Powell-Eyring fluid flow due to a stretching sheet with convective boundary conditions and slip velocity

**DOI:** 10.1038/s41598-023-42609-w

**Published:** 2023-09-21

**Authors:** W. Abbas, Ahmed M. Megahed, M. S. Emam, Hassan M. H. Sadek

**Affiliations:** 1 Basic and Applied Science Department, College of Engineering and Technology, Arab Academy for Science, Technology and Maritime Transport, Cairo, Egypt; 2 Department of Mathematics, Faculty of Science, Benha University, Benha, Egypt; 3 Physics and Engineering Mathematics Department, Faculty of Engineering-Mattaria, Helwan University, Cairo, Egypt

**Keywords:** Engineering, Mechanical engineering, Applied mathematics

## Abstract

The novelty and motivation of this research can be emphasized by examining how the heat transfer mechanism of a non-Newtonian Powell-Eyring fluid, which flows because of a stretched sheet, is affected by factors like viscous dissipation, the slip velocity phenomenon, and Joule heating. In addition, the investigation delves into the heat transfer behavior of the fluid flow when it comes into contact with a convectively heated stretched surface that is influenced by varying fluid properties. This analysis also takes into account the influence of changing fluid characteristics and the presence of magnetic field. The numerical solutions of modelled equations that governing the problem are detected using the shooting technique. Also, in order to confirm the validity of the present investigation, a proper comparison with certain published works as a particular case of the present model is presented, and a perfect agreement is noted. With the use of diagrams and tables, the flow problem’s effective parameters are thoroughly discussed. Likewise, through a tabular representation, the values of the local Nusselt number and the skin-friction coefficient are computed and analyzed. Many significant conclusions can be drawn from numerical results. Most importantly, the local Nusselt number rises monotonically with both the surface convection parameter and the slip velocity parameter, but the local skin-friction coefficient has the opposite trend. The results indicate that the nanofluid temperature is enhanced by factors such as the surface convection parameter, magnetic field, and viscous dissipation. On the other hand, the slip velocity phenomenon leads to the opposite effect.

## Introduction

Monitoring flow of fluids over stretching surfaces and investigation of their behavior recently occupied a significant regard between researchers in engineering and science aspects due to their major role in several of industrial applications such as plastic manufacturing and rubber as well, photographic films coating by liquids and manufacturing of lubricants etc.^1^. Beginning with the pioneer Sakiadis^2^ studied Blasius type flow over a stretching sheet where the speed was constant and the fluid at rest. Sequentially Crane^3^ investigated the flow of fluid which has common characteristics with boundary layer of tile Hiemenze flow and the velocity of stretching sheet is proportional to distance of the slit. The analytical solution of flow past stretching surface in the existence of magnetic field in two dimensions was studied by Kumari and Nath^4^. Abel et al.^5^ investigated the transfer of the heat in visco-elastic fluid through porous medium over surface where the viscosity was variable with temperature. Sanjayanand et al.^6^ obtained the analytical solution of zeroth order of stream function. The Mass and heat transfer equations are investigated past continuous stretching sheet to obtain the hypergeometric solutions. Chamkha and Mansour^7^ studied the transfer of heat past a stretching sheet in the presence of chemical reaction through porous medium and the unsteady free convection as well. The influence of temperature and space on flow which was generated over a stretching surface in the existance of normally applied magnetic field was studied by Ganga et al.^8^. The generation of entropy on steady incompressible Carreau liquid flow past a stretching sheet with curved shape in two dimensions was studied by Raza et al.^9^. Megahed et al.^10^ studied the MHD fluid flow with heat transfer past an unsteady stretching sheet under thermal radiation and heat flux effects. Tawade et al.^11^ obtained the solution of nonlinear ordinary differential equations using the approach of Runge-Kutta 4th-order with shooting for nanofluid flow which was laminar and unsteady past stretching surface in two dimensions. Some further problems related to the fluid flow under to a stretchable sheet with variables thickness for different conditions can be found in the literature^12–14^. Later, there are numerous researchers development about nanofluids flow subject to stretchable sheets are highlighted in Refs.^15–17^.

Over the years, many researchers have aimed to study the properties of fluids, especially non-Newtonian fluids, which have become extremely popular in various industrial, bio-fluid, foods, and medical fields^18–20^. Powell-Eyring model is one of the non-Newtonian fluid models which is derived from kinetic theory of gases instead of empirical formula. Many researchers used the Powell-Eyring model under different physical conditions. Hayat et al.^21^ obtained exact solution of magnetohydrdynamic eyring powell fluid flow past a stretching surface and discussed radiative effect as well. The analytic solution for the Powell-Erying fluid flow past a nonlinear stretching sheet was investigated by Panigrahi et al.^22^. Gaffar et al.^23^ investigated the Eyring-Powell fluid flow over vertical surface through porous medium. Hayat et al.^24^ studied flow of Eyring-Powell fluid past an exponentially stretching surface in the Prescence of chemical reactions and heat flux. The influence of convective boundary conditions, viscous dissipation and activation energy on Eyring Powell nanofluid through porous medium was examined by Nazeer et al.^25^. Patil et al.^26^ studied the effect of chemical reaction and thermal radiation of unsteady Nano Powell-Eyring fluid flow behind stagnation point. Khader and Babatin^27^ carried out a numerical solution by using the spectral collocation method the flow of Powell-Eyring fluid in the existence of magnetic field and thermal radiation.

It’s important to note that earlier research overlooked the heat transfer process of a Powell-Eyring fluid, which is impacted by elements such as viscous dissipation, slip velocity phenomenon, and Joule heating. Additionally, prior studies did not consider a convectively heated stretched surface that is influenced by changing fluid properties, all within the same investigation. Consequently, the novelty and impetus behind the present research are driven by insights from the literature review, urging an exploration into the impacts of some important phenomena like viscous dissipation, slip velocity, Joule heating, and magnetic field on the flow behavior of the convectively heated Powell-Eyring fluid. Notably, this fluid’s properties undergo thermal changes due to the stretching sheet.

## Analysis of the problem

**Figure 1:**
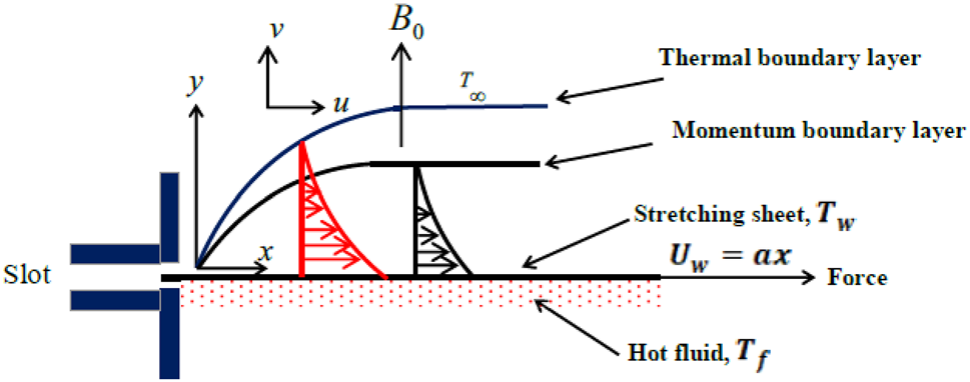
Physical configuration.

Consider a Powell-Eyring fluid moving as indicated in Fig. 1 due to a rough stretching sheet. Owing to the shear, the fluid moved slowly, and its velocity had the form $U_{w}=ax$. External magnetic fields with strength *B* are applied to fluid layers, causing them to impart in the transverse direction. In our research, we suppose that a hot fluid exists beneath the stretching sheet’s bottom surface.

This hot fluid is vital in heating the surface of the stretched sheet by convection with the temperature $T_{f}=T_{\infty }+Ax^{m}$, where $T_{\infty }$ which is invariably less than $T_{f}$ corresponds to a constant cold fluid temperature away from the sheet, *A* and *m* are constants. The viscous dissipation phenomena is also considered for a better understanding of heat transfer mechanism. The energy equation takes into account the effect of a Joule heating. The rules for fluid and heat transport are determined by the foregoing axioms and the usual Boussinesq’s approximation ^1^:

1 $$\begin{aligned}{} & {} \frac{\partial u}{\partial x}+\frac{\partial v}{\partial y}=0, \end{aligned}$$

2 $$\begin{aligned}{} & {} u\frac{\partial u}{\partial x}+v\frac{\partial u}{\partial y}=\frac{1}{\rho _{\infty }}\frac{\partial }{\partial y} \Bigg ( \mu \frac{\partial u}{\partial y}+ \frac{1}{\tilde{\beta }C}\frac{\partial u}{\partial y}-\frac{1}{6\tilde{\beta }C^{3}} \left( \frac{\partial u}{\partial y}\right) ^{3}\Bigg )-\frac{\sigma B^{2}}{\rho _{\infty }}u, \end{aligned}$$

3 $$\begin{aligned}{} & {} u\frac{\partial T}{\partial x}+v\frac{\partial T}{\partial y} =\frac{1}{\rho _{\infty } c_{p}}\frac{\partial }{\partial y}\Big (\kappa \frac{\partial T}{\partial y}\Big )+\frac{\sigma B^{2}}{\rho _{\infty }c_{p}}u^{2}+\frac{1}{\rho _{\infty } c_{p}}\Bigg ( \mu \left( \frac{\partial u}{\partial y}\right) ^{2}+ \frac{1}{\tilde{\beta }C} \left( \frac{\partial u}{\partial y}\right) ^{2}-\frac{1}{6\tilde{\beta }C^{3}} \left( \frac{\partial u}{\partial y}\right) ^{4}\Bigg ), \end{aligned}$$

where the components of velocity are denoted by (*u*, *v*), $\rho _{\infty }$ is the ambient fluid density, the symbol $\sigma$ represents the ability of a powell-Eyring fluid to conduct electricity and $c_{p}$ is the specific heat. The Joule effect occurs as electric current passes through a conductive liquid, causing heat due to its inherent resistance. In the context of fluid flow over a horizontally stretched sheet, the presence of an electric field or current can induce Joule heating. The fluid thermal conductivity is defined by $\kappa$, whereas its viscosity is symbolized by $\mu$. Further, $\tilde{\beta }$ and *C* are the Powell-Eyring fluid parameters. These parameters ($\tilde{\beta }$ and *C*) are highly significant, as they control the distinct rheological characteristics and behavior of this non-Newtonian material. They directly influence how the fluid behaves and interacts in various situations, offering valuable insights into its response to external factors. Adding slip velocity to the velocity field’s condition adds to the originality. The continuity, momentum, and energy equations are all linked to the following boundary conditions ^28^:

4 $$\begin{aligned}{} & {} u=ax+\frac{\lambda _{1}}{\mu _{\infty }} \Bigg (\mu \frac{\partial u}{\partial y}+ \frac{1}{\tilde{\beta }C}\frac{\partial u}{\partial y}-\frac{1}{6\tilde{\beta }C^{3}} \left( \frac{\partial u}{\partial y}\right) ^{3}\Bigg ), \quad v=0, \quad -\kappa \Bigg (\frac{\partial T}{\partial y}\Bigg )=h_{f} \left( T_{f}-T_{w}\right) \quad at \quad y=0, \end{aligned}$$

5 $$\begin{aligned}{} & {} u\rightarrow 0,\hspace{0.3 cm} T\rightarrow T_{\infty } \hspace{0.3 cm} as \hspace{0.2 cm} y\rightarrow \infty . \end{aligned}$$

The following is a list of recommended dimensionless variables ^29^:

6 $$\begin{aligned} \psi= & {} \sqrt{a\nu _{\infty }}x f(\eta ),\quad \quad \eta = \sqrt{\frac{a}{\nu _{\infty }}}y, \end{aligned}$$

7 $$\begin{aligned} \theta (\eta )= & {} \frac{T-T_{\infty }}{T_{f}-T_{\infty }}. \end{aligned}$$

In light of the previously mentioned dimensionless variables, the governing equations are rewritten in the following way:

8 $$\begin{aligned}{} & {} f'''\Big (e^{-\gamma \theta }+\alpha (1-\delta f''^{2})\Big )-\gamma f'' \theta 'e^{-\gamma \theta }-f'^{2}+ff''-M f'=0, \end{aligned}$$

9 $$\begin{aligned}{} & {} \frac{1}{Pr}\left( \left( 1+\varepsilon \theta \right) \theta ''+\varepsilon \theta '^{2}\right) +f\theta '-2f'\theta + Ecf''^{2}\left[ \left( e^{-\gamma \theta }+\alpha \right) -\frac{\alpha \delta }{3}f''^{2} \right] +M Ec f'^{2}=0, \end{aligned}$$

10 $$\begin{aligned}{} & {} f(0)=0,\quad f'(0)=1+\lambda \left( \left( e^{-\gamma \theta (0)}+\alpha \right) f''(0)-\frac{\alpha \delta }{3}f''^{3}(0) \right) ,\quad \theta '(0)=-\Delta \left( \frac{1-\theta (0)}{1+\varepsilon \theta (0)}\right) , \end{aligned}$$

11 $$\begin{aligned}{} & {} f'\rightarrow 0,\quad \theta \rightarrow 0, \quad as \quad \eta \rightarrow \infty , \end{aligned}$$

where a prime denotes differentiation with respect to $\eta$. Also, the yielded parameters $\alpha , \delta$ represents the dimensionless material fluid parameters, $\lambda$ denotes the slip parameter, *M* is the magnetic parameter, $\Delta$ denotes the surface-convection parameter, *Ec* symbolize to the Eckert number and *Pr* is the Prandtl number. All of these parameters can be described as follows:

12 $$\begin{aligned} \alpha= & {} \frac{1}{\mu _{\infty } \tilde{\beta } C}, \quad \delta =\frac{a \rho _{\infty } U_{w}^{2}}{2 \mu _{\infty } C^{2}}, \quad \lambda =\lambda _{1}\sqrt{\frac{a}{\nu _{\infty }}}, \quad M=\frac{\sigma B^{2}}{a \rho _{\infty }}, \end{aligned}$$

13 $$\begin{aligned} \Delta= & {} \frac{h_{f}}{\kappa _{\infty }}\sqrt{\frac{\nu _{\infty }}{a}} \quad Ec=\frac{a^{2}}{A c_{p}}, \quad Pr=\frac{\mu _{\infty } c_{p}}{\kappa _{\infty }}. \end{aligned}$$

Furthermore, identifying the features for both temperature and velocity fields that can be investigated after focusing on obtaining the numerical solution for the proposed model may have aided us in calculating the values of the local skin-friction coefficient $Cf_{x}$ and the local Nusselt number $Nu_{x}$ as follows:

14 $$\begin{aligned} Cf_{x}Re^{\frac{1}{2}}=-\left[ \left( e^{-\gamma \theta (0)}+\alpha \right) f''(0)-\frac{\alpha \delta }{3}f''^{3}(0) \right] ,\quad Nu_{x}Re^{\frac{-1}{2}}=-\theta '(0), \end{aligned}$$

where $Re=\frac{U_{w} x}{\nu _{\infty }}$ is the local Reynolds number.

## Validation of numerical scheme

To support the numerical calculations performed in this work, the results of this study were compared to those published in one of the famous journals, which is addressed further down by Hayat et al.^29^. The previous work of Hayat et al.^29^, which was performed with the homotopy analysis method, can be compared with our current work in the absence of $M, \gamma ,$ and $\lambda$. The variation in the results, as shown in Table 1, is quite small, with a maximum variance of 0.000005 in $f''(0)$ values, showing that the solution used in this study is valid. The new results reported here are dependable and very accurate to a reasonable degree.

**Table 1:** Computed values of $-f''(0)$ for different values of $\alpha$ and $\delta$ when $M=\gamma =\lambda =0$.

$\alpha$	$\delta$	Hayat et al.^29^	Present work
0.1	0.1	0.956018	0.956016949
0.2	0.1	0.917972	0.917971807
0.3	0.1	0.883221	0.883219986
0.1	0.1	0.956018	0.956016949
0.1	0.5	0.964859	0.964858906
0.1	1.0	0.975361	0.975310998

## Results and discussion

**Figure 2:**
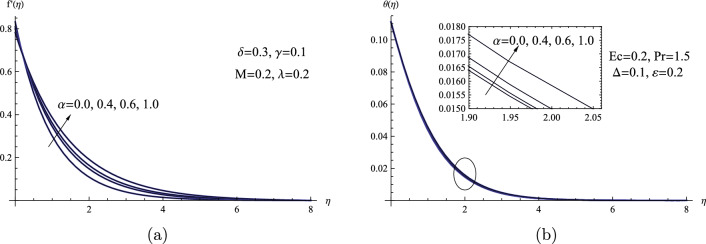
(**a**) $f'(\eta )$ for various values of $\alpha$. (**b**) $\theta (\eta )$ for various values of $\alpha$.

This portion of the discussion uses many variables that correspond to the flow under consideration to show physical descriptions of velocity and temperature profiles. Thus, Runge-Kutta of order four is used in conjunction with the shooting technique in Mathematica software. We found that the calculated results had significant impacts. Figure 2 shows a schematic illustration of the $\alpha$ parameter and its impact on both the velocity $f'(\eta )$ and temperature $\theta (\eta )$ fields. The velocity distribution $f'(\eta )$ is greatly improved by increasing the value of the $\alpha$ parameter, although the temperature distribution $\theta (\eta )$ is only somewhat improved. Physically, the Powell-Eyring parameter $\alpha$ drives a rise in fluid velocity due to its reciprocal connection with fluid viscosity. When the Powell-Eyring parameter increases, fluid viscosity decreases, reducing internal resistance. This facilitates freer movement of fluid molecules, leading to a noticeable increase in velocity.

**Figure 3:**
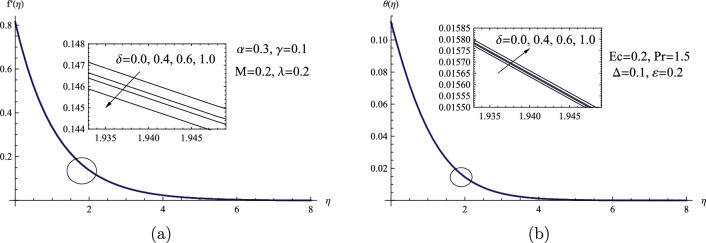
(**a**) $f'(\eta )$ for various values of $\delta$. (**b**) $\theta (\eta )$ for various values of $\delta$.

In Fig. 3, predictions of the velocity distribution $f'(\eta )$ and the temperature distribution $\theta (\eta )$ versus the dimensionless variable $\eta$ for the $\delta$ parameter are displayed. Clearly that, the $\delta$ parameter’s numerical value can be increased to slightly reduce the speed of the Powell-Eyring fluid flow, whereas the reverse trend can be achieved for the temperature distribution for the same parameter $\delta$.

**Figure 4:**
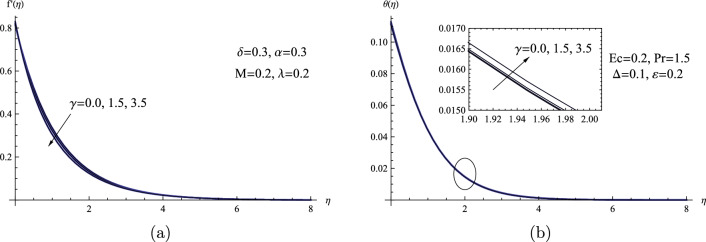
(**a**) $f'(\eta )$ for various values of $\gamma$. (**b**) $\theta (\eta )$ for various values of $\gamma$.

Figure 4 displays the values of $\gamma$ acquired in the current study that describe the viscosity of the Powell-Eyring fluid and how it affects both flow and the heat transfer mechanism. The viscosity parameter is connected with an increased shear stress of Powell-Eyring fluid, raising the fluid viscosity, so it is evident that increasing the values of the viscosity parameter diminish fluid velocity. Physically, the viscosity parameter adds internal resistance to the fluid’s molecular arrangement, leading to an internal friction effect. With higher viscosity, the interactions between the fluid’s molecules become more significant, impeding their unrestricted motion. As a result, this heightened internal resistance causes a slight reduction in the fluid’s overall speed, as the fluid particles encounter more resistance during their flow. On the other hand, as can be seen from Fig. 4b, the viscosity parameter also somewhat increases the temperature field.

**Figure 5:**
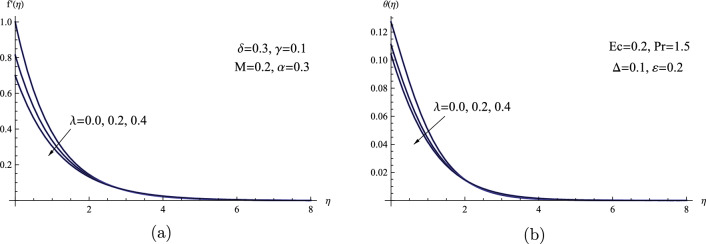
(**a**) $f'(\eta )$ for various values of $\lambda$. (**b**) $\theta (\eta )$ for various values of $\lambda$.

Figure 5 illustrates the significance of the slip velocity phenomena on the flow and heat transfer rate as well as the velocity distribution. It has been observed that both the temperature profiles $\theta (\eta )$ and the velocity profiles $f'(\eta )$ decrease as the slip velocity parameter’s value is increased. Physically, this phenomenon can be attributed to the slip velocity effect, which is believed to indicate the presence of irregularities or it reflect the existence of roughness on the surface of the sheet. Consequently, this particular parameter acts to decelerate the fluid velocity within the boundary layer region.

**Figure 6:**
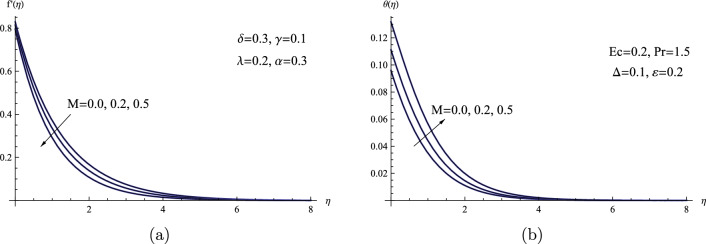
(a) $f'(\eta )$ for various values of *M*.(b) $\theta (\eta )$ for various values of *M*.

Figure 6 shows ways for producing heat and controlling the velocity field through the influence of the magnetic field on Powell-Eyring fluid flow within the boundary layer. As growing values of magnetic parameter *M* are directly related to the generation of increased resistance force, also known as Lorentz force, it acts in a manner perpendicular to the path of fluid flow; hence, as Lorentz force increases, impedance force also increases and attempts to oppose fluid flow, thinning the thickness of the boundary layer. It follows naturally that raising the magnetic parameter improves the temperature of the sheet $\theta (0)$ and the temperature distribution. As a consequence, contributes to increasing the thermal boundary layer’s thickness. Furthermore, the influence of the magnetic field on both the flow dynamics and heat transfer mechanisms can be substantiated by referring to the significant and relevant studies that were previously presented in the literature review^30^–^33^. This lends further support to the understanding of how the magnetic field impacts these processes.

**Figure 7:**
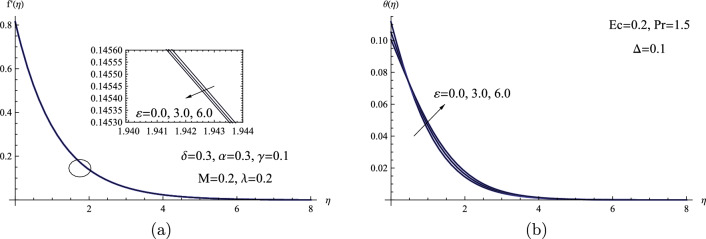
(**a**) $f'(\eta )$ for various values of $\varepsilon$. (**b**) $\theta (\eta )$ for various values of $\varepsilon$.

Photographs of the temperature and velocity patterns in relation to the thermal conductivity parameter $\varepsilon$ are shown in Fig. 7. As a result of the thermal conductivity parameter’s indirect influence on the fluid velocity field, a minor reduction in fluid velocity distribution is seen. Also, a higher thermal conductivity parameter implies a rise in the molecules kinetic energy, which causes more collisions between molecules and, as a result, a higher temperature distribution within the thermal region away from the sheet.

**Figure 8:**
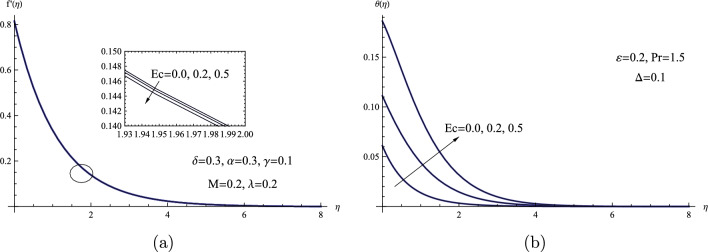
(**a**) $f'(\eta )$ for various values of *Ec*. (**b**) $\theta (\eta )$ for various values of *Ec*.

Figure 8 depicts the fluid flow’s temperature and velocity distributions in the boundary layer medium over a range of Eckert number values *Ec*. The Eckert number *Ec* represents the transformation of kinetic energy into stored energy, which is then dispersed as heat, through work performed versus the stresses of the viscous fluid. Thus, an enhancement in the sheet temperature $\theta (0)$ and the temperature distribution is caused by higher viscous heat release. For the velocity distribution, a very slight reverse trend was seen. Physically, the Joule effect emerges as electric current moves through a conductive fluid, producing heat due to its natural resistance. In the context of fluid flow over a stretched sheet, an electric field or current can cause Joule heating. This is often included in the energy equation to handle heat generated by the interaction of the electric field and fluid’s conductivity. The resulting temperature rise from Joule heating could impact the flow’s overall heat transfer characteristics.

**Figure 9:**
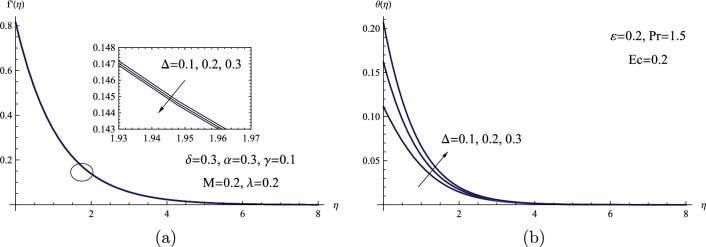
(**a**) $f'(\eta )$ for various values of $\Delta$. (**b**) $\theta (\eta )$ for various values of $\Delta$.

The distributions of velocity and temperature are each subject to the influence of the surface-convection parameter $\Delta$, as shown in Fig. 9. Due to high surface-convection parameter $\Delta$, both the sheet temperature $\theta (0)$ and the fluid temperature increase because the surface-convection parameter is directly proportional to the heat transfer coefficient. Also, with higher values of the same parameter, the velocity distribution within the boundary layer marginally decreased. Physically, the surface-convection parameter improves the heat exchange at the fluid’s surface, causing a more rapid transfer of heat from the fluid to its surroundings. With an increase in the surface-convection parameter, this enhanced heat transfer leads to a temperature increase in the fluid. In summary, a higher surface-convection parameter facilitates more efficient heat dissipation from the fluid, leading to a noticeable temperature rise.

As shown in Table 2, the skin friction coefficient for Powell-Eyring fluid explicitly rises with the magnetic parameter and thermal conductivity parameter but diminishes with the surface-convection parameter and Eckert number. Skin-friction coefficients are reduced in magnitude due to the effect of the slip velocity parameter. Physically, the greater slip velocity parameter produces more resistance to fluid movement, which has the effect of reducing the shear stresses at the sheet’s surface. Local Nusselt number is seen to be rising in correlation with both slip velocity and surface-convection parameter values. Additionally, the thermal conductivity parameter, Eckert number, magnetic number, and viscosity parameter are all improved in order to perceive the Nusselt number’s lowering behavior.

**Table 2:** Values of $Cf_{x}(Re_{x})^{\frac{1}{2}}$ and $Nu_{x}Re_{x}^{\frac{-1}{2}}$ for different values of $\alpha , \delta , \gamma , \lambda$, *M*, $\varepsilon$, *Ec* and $\Delta$ with $Pr=1.5$.

$\alpha$	$\delta$	$\gamma$	$\lambda$	*M*	$\varepsilon$	*Ec*	$\Delta$	$Cf_{x}Re_{x}^{\frac{1}{2}}$	$Nu_{x}Re_{x}^{\frac{-1}{2}}$
0.0	0.3	0.1	0.2	0.2	0.2	0.2	0.1	0.841337	0.0869572
0.4	0.3	0.1	0.2	0.2	0.2	0.2	0.1	0.955088	0.0869536
0.6	0.3	0.1	0.2	0.2	0.2	0.2	0.1	1.003871	0.0869525
1.0	0.3	0.1	0.2	0.2	0.2	0.2	0.1	1.089652	0.0869522
0.3	0.0	0.1	0.2	0.2	0.2	0.2	0.1	0.931338	0.0869651
0.3	0.4	0.1	0.2	0.2	0.2	0.2	0.1	0.928037	0.0869509
0.3	0.6	0.1	0.2	0.2	0.2	0.2	0.1	0.926329	0.0869434
0.3	1.0	0.1	0.2	0.2	0.2	0.2	0.1	0.922785	0.0869276
0.3	0.3	0.0	0.2	0.2	0.2	0.2	0.1	0.931092	0.0869608
0.3	0.3	1.5	0.2	0.2	0.2	0.2	0.1	0.898021	0.0868687
0.3	0.3	3.5	0.2	0.2	0.2	0.2	0.1	0.854723	0.0867553
0.3	0.3	0.1	0.0	0.2	0.2	0.2	0.1	1.236590	0.0851187
0.3	0.3	0.1	0.2	0.2	0.2	0.2	0.1	0.928876	0.0869545
0.3	0.3	0.1	0.4	0.2	0.2	0.2	0.1	0.752135	0.0877525
0.3	0.3	0.1	0.2	0.0	0.2	0.2	0.1	0.855818	0.0887036
0.3	0.3	0.1	0.2	0.2	0.2	0.2	0.1	0.928876	0.0869545
0.3	0.3	0.1	0.2	0.5	0.2	0.2	0.1	1.023750	0.0845998
0.3	0.3	0.1	0.2	0.2	0.0	0.2	0.1	0.928873	0.0888348
0.3	0.3	0.1	0.2	0.2	3.0	0.2	0.1	0.928918	0.0680239
0.3	0.3	0.1	0.2	0.2	6.0	0.2	0.1	0.929906	0.0560986
0.3	0.3	0.1	0.2	0.2	0.2	0.0	0.1	0.930068	0.0927649
0.3	0.3	0.1	0.2	0.2	0.2	0.2	0.1	0.928876	0.0869545
0.3	0.3	0.1	0.2	0.2	0.2	0.5	0.1	0.927093	0.0784689
0.3	0.3	0.1	0.2	0.2	0.2	0.2	0.1	0.928876	0.0869545
0.3	0.3	0.1	0.2	0.2	0.2	0.2	0.2	0.928021	0.1623830
0.3	0.3	0.1	0.2	0.2	0.2	0.2	0.3	0.927259	0.2284992

## Main remarks

A computational model that incorporates viscous dissipation and convective boundary conditions for a slippery, non-Newtonian Powell-Eyring fluid flowing toward a stretching surface has been interpreted. A set of nonlinear ordinary differential equations is produced by incorporating an appropriate dimensionless transformation into the governing model. Significant data are discussed here after hiring shooting approach to analyze the fluid velocity, fluid temperature, the local skin-friction, and Nusselt number. Following are some conclusions that can be derived from the findings of this study:

1. The slip velocity phenomenon weakened the velocity, temperature distributions, and the skin-friction coefficient, in particular.
2. As the magnetic number or the surface convection parameter rises, both the sheet temperature and the temperature distribution within the thermal boundary region rise as well.
3. As the dimensionless material fluid parameters or the viscosity parameter increases, the rate of heat transfer at the wall decreases.
4. Higher conductivity values result in a rise in the temperature profile, however a reversal of this pattern can be seen beside the sheet.
5. By increasing the viscosity parameter’s value, the liquid’s velocity decreases while the temperature field increases slightly.
6. In future research, we aim to build upon this study by exploring heat and mass fluxes. The goal is to regulate the cooling process utilizing nanofluids.

## Data Availability

The datasets used and/or analyzed during the current study available from the corresponding author on reasonable request.

## List of symbols

*a*: Coefficient of velocity $\left( \textrm{s}^{-1}\right)$
*B*: Intensity of a magnetic field (T)
*C*: Material parameter of Eyring-Powell model $\left( \textrm{s}^{-1}\right)$
$Cf_{x}$: Skin friction coefficient
$c_{p}$: Heat capacity under constant pressure $\left( \mathrm{J \,\,kg}^{-1} \textrm{K}^{-1}\right)$
*Ec*: Eckret number
*f*: Dimensionless stream function
$h_{f}$: Thermal flux coefficient $\left( \mathrm{W \,\,m}^{-2} \textrm{K}^{-1}\right)$
*M*: Magnetic parameter
$Nu_{x}$: Local Nusselt number
*Pr*: Prandtl number
*Re*: Local Reynolds number
*T*: Powell-Eyring temperature (K)
$$T_{f}$$: Convection temperature (K)
$T_{\infty }$: Temperature away the sheet (K)
*u*: Velocity element along the $x-$axis $\left( \mathrm{m \,\,s}^{-1}\right)$
$U_{w}$: Velocity of the stretching sheet $\left( \mathrm{m \,\,s}^{-1}\right)$
*v*: Velocity element along the $y-$axis $\left( \mathrm{m \,\,s}^{-1}\right)$
*x*, *y*: Cartesian coordinates (m)

## Greek symbols

$$\mu $$: Viscosity coefficient $$\left( \mathrm{kg \,\,m}^{-1} s^{-1}\right) $$
$\mu _{\infty }$: Viscosity of Powell-Eyring fluid at infinity $\left( \mathrm{kg \,\,m}^{-1} \textrm{s}^{-1}\right)$
$$\sigma $$: Electrical conductivity $$\left( \mathrm{S \,\,m}^{-1}\right) $$
$$\nu $$: Kinematic viscosity $$\left( \textrm{m}^{2}\textrm{s}^{-1}\right) $$
$\nu _{\infty }$: Kinematic viscosity away the sheet $\left( \textrm{m}^{2}\textrm{s}^{-1}\right)$
$\rho$: Density of fluid $\left( \mathrm{kg \,\,m}^{-3}\right)$
$\rho _{\infty }$: Density away the sheet $\left( \mathrm{kg \,\,m}^{-3}\right)$
$\tilde{\beta }$: Material parameter of Eyring-Powell model $\left( \mathrm{m \,\,s}^{2}\textrm{kg}^{-1}\right)$
$\eta$: Dimensionless similarity variable
$\lambda$: Slip velocity parameter
$\lambda _{1}$: Slip velocity factor (m)
$$\gamma $$: Viscosity parameter
$\alpha , \delta$: Dimensionless Eyring-Powell parameters
$$\psi $$: Stream function $$\left( \textrm{m}^{2}\textrm{s}^{-1}\right) $$
$\Delta$: Surface-convection parameter
$$\theta $$: Dimensionless temperature
$\varepsilon$: Thermal conductivity parameter
$$\kappa $$: Thermal conductivity $$\left( \mathrm{W \,\,m}^{-1}\textrm{K}^{-1}\right) $$
$\kappa _{\infty }$: Thermal conductivity away the sheet $\left( \mathrm{W \,\,m}^{-1}\textrm{K}^{-1}\right)$

## Superscripts

$\prime$: Differentiation with respect to $\eta$
*w*: Wall condition
$\infty$: Free stream condition
